# A global repository of novel antimicrobial emergence events

**DOI:** 10.12688/f1000research.26870.2

**Published:** 2021-06-21

**Authors:** Emma Mendelsohn, Noam Ross, Allison M. White, Karissa Whiting, Cale Basaraba, Brooke Watson Madubuonwu, Erica Johnson, Mushtaq Dualeh, Zach Matson, Sonia Dattaray, Nchedochukwu Ezeokoli, Melanie Kirshenbaum Lieberman, Jacob Kotcher, Samantha Maher, Carlos Zambrana-Torrelio, Peter Daszak

**Affiliations:** 1 EcoHealth Alliance, New York, NY, 10018, USA; 2 Current Address: Office of U.S. Foreign Disaster Assistance, USAID, District of Columbia, USA; 3 Current Address: Department of Epidemiology and Biostatistics, Memorial Sloan Kettering Cancer Center, New York, NY, USA; 4 Current Address: Mailman School of Public Health, Columbia University, New York, NY, USA; 5 Current Address: American Civil Liberties Union, New York, NY, USA; 6 Current Address: Biology Department, Graduate Center of the City University of New York, New York, NY, USA; 7 Current Address: Oak Ridge Institute for Science and Education Fellow, Centers for Disease Control and Prevention, Atlanta, GA, USA; 8 Current Address: Health Union, Philadelphia, PA, USA; 9 Current Address: Guidehouse, Chicago, IL, USA; 10 Current Address: School of Veterinary Medicine, University of Pennsylvania, Philadelphia, PA, USA; 11 Current Address: Department of Geoscience, Hobart and William Smith Colleges, Geneva, NY, USA

**Keywords:** Antimicrobial resistance, global health, open-access data

## Abstract

Despite considerable global surveillance of antimicrobial resistance (AMR), data on the global emergence of new resistance genotypes in bacteria has not been systematically compiled. We conducted a study of English-language scientific literature (2006-2017) and ProMED-mail disease surveillance reports (1994-2017) to identify global events of novel AMR emergence (first clinical reports of unique drug-bacteria resistance combinations). We screened 24,966 abstracts and reports, ultimately identifying 1,757 novel AMR emergence events from 268 peer-reviewed studies and 26 disease surveillance reports (294 total). Events were reported in 66 countries, with most events in the United States (152), China (128), and India (127). The most common bacteria demonstrating new resistance were *Klebsiella pneumoniae* (344) and *Escherichia coli *(218). Resistance was most common against antibiotic drugs imipenem (89 events), ciprofloxacin (84) and ceftazidime (83). We provide an open-access database of emergence events with standardized fields for bacterial species, drugs, location, and date. We discuss the impact of reporting and surveillance bias on database coverage, and we suggest guidelines for data analysis. This database may be broadly useful for understanding rates and patterns of AMR evolution, identifying global drivers and correlates, and targeting surveillance and interventions.

## Introduction

Antimicrobial resistance (AMR) is a global health crisis that has compromised the effective treatment and prevention of a multitude of infections. The rise in AMR has been associated with increased mortality, longer hospitalizations, complications with medical procedures such as surgery and chemotherapy, and higher healthcare costs ^1– 3^. Resistance to antibiotics is a global public health issue and a particular concern in low- and middle-income countries, where many high-burden diseases such as malaria, respiratory infections, and tuberculosis can no longer be treated by common antimicrobial drugs ^1, 4^. Combating AMR requires a multidimensional global response to optimize antimicrobial drug use, improve awareness, increase traceability and usage reporting, and promote research ^1, 5– 7^.

AMR surveillance by researchers, hospital networks, and state governments is key to characterizing and responding to the crisis. Current global-scale datasets primarily focus on the presence or prevalence of known resistance genotypes and phenotypes in bacterial populations. In 2014, the World Health Organization (WHO) published surveillance data obtained from 129 member states on nine bacterial pathogen-antibacterial drug combinations of public health importance, finding high rates of resistance reported across the globe ^3^. The ResistanceMap database, maintained by the Center of Disease Dynamics, Economics & Policy, provides nationally-aggregated data from 46 countries on the prevalence of resistance in 12 bacterial species against 17 classes of antibiotics ^8^.

To our knowledge, however, there is no publicly available dataset that specifically identifies the spatial and temporal patterns of the emergence of novel bacterial pathogen resistance to antibiotic drugs in humans. Such data are critical to understanding macroecological patterns and drivers of AMR emergence and identifying geographic and phenotypic targets for surveillance, research, and interventions. This repository is relevant to the development of country-specific National Action Plans (NAPs) to reduce the emergence and spread of AMR, as well as to on-going agreements, programs and priorities for intergovernmental organizations (e.g., Tripartite-Plus Alliance on AMR, FAO, OIE, UNEP and WHO) and multi-lateral development banks and financing facilities (e.g. World Bank). In addition, a better understanding of factors that contribute to AMR emergence may bolster the work of the Interagency Coordination Group (IACG) on Antimicrobial Resistance (convened by the UN Secretary General) to target drivers of AMR that are important for orientation and collective action ^7^.

We conducted a systematic review to create a repository of novel AMR emergence events reported in English-language scientific literature from 2006–2017 and ProMED-mail disease surveillance reports from 1994–2017. We focused specifically on human clinical cases and did not limit the search to any subset of bacteria or drugs. We screened 24,966 abstracts and reports, identifying 1,791 articles potentially reporting novel emergence events, and ultimately, 294 relevant articles reporting 1,757 total novel events. We present an open-access database of these events with cleaned and standardized fields for location of emergence, bacterial species, antimicrobial drug, event start date, and data source. We provide usage notes on systematic biases and ambiguities in the data. In addition, we provide data on all screened and processed articles from which data were harvested.

## Methods

**Figure 1.**
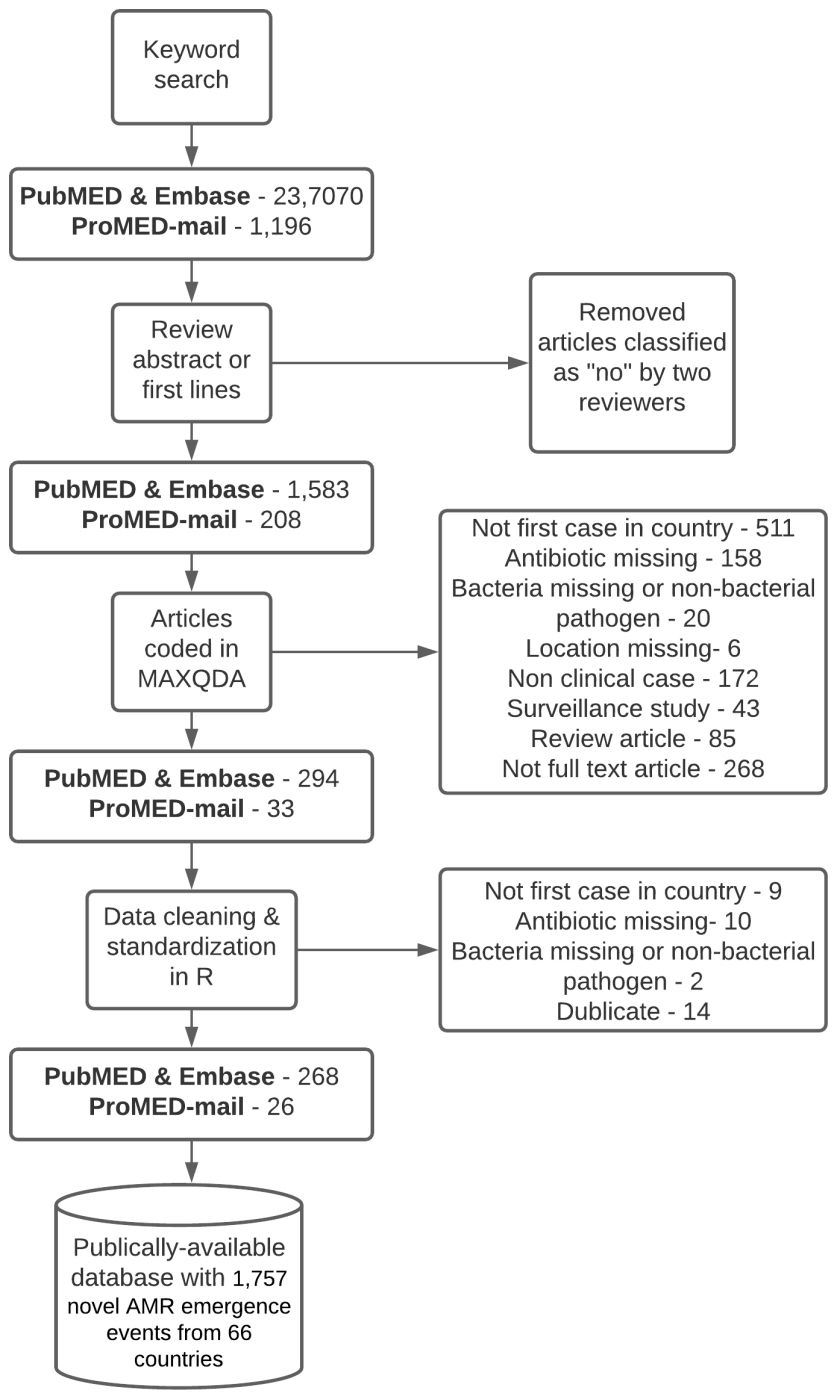
Prisma flow diagram, showing the workflow of information through steps of the systematic review. Note some articles were excluded based on more than one criteria.

Development of the AMR emergence database was a multi-step process consisting of a systematic literature search, abstract and report screening, article review and coding, and data cleaning and standardization ( Figure 1).

### Literature search

We drew from PubMed and Embase scientific literature databases and ProMED-mail to develop our database of events. PubMed and Embase collectively encompass a large fraction of peer-reviewed, English-language biomedical scientific literature, while ProMED-mail consists of clinical reports and news alerts that have a faster reporting speed and may not ultimately be published in the scientific press. We did not assess quality of studies or reports; however, we indicate in the database whether an event is from a ‘peer-reviewed study’ or ‘ProMED-mail report’.

We searched for a recent ten-year period for scientific literature (2006–2017). ProMED-mail reports were drawn from a longer time span (1994–2017), as we found scientific articles published 2006–2017 frequently described events occurring many years before.

We searched for manuscripts in PubMed and Embase using the following terms:

*'antibiotic resistance'/exp OR ('antibiotic' AND ('resistant' OR 'resistance')) OR 'antimicrobial resistance'/exp OR ('antimicrobial' AND ('resistant' OR 'resistance')) AND (first OR novel OR new OR emerging OR emergent) AND (case OR patient) AND [humans]/lim AND [2006–2017]/py*

This search, completed August 2018, yielded 23,770 results.

Because ProMED-mail searches use partial word stem-based matching, we used only the search terms ‘ *antibiotic resistance’* and *'antimicrobial resistance’*, which yielded 1,196 results.

A total of 24,966 articles (peer-reviewed studies and ProMED-mail reports) were compiled for screening.

### Abstract and report screening

We used the following inclusion criteria to screen the results of our literature search: the article must have described at least one **clinical case** (a) of infectious disease in a **patient** (b) caused by a **novel** (c) **resistance** (d) to a particular **antimicrobial drug or drug combination** (e) in **bacteria** (f). In this definition:

- **a)** A “clinical case” is an individual who presents with symptoms to a medical professional and is determined by a medical professional to have been infected with the bacterium species reported. Antimicrobial resistance in an asymptomatic individual’s commensal bacteria—as determined by a screening study, a “challenge” study, or a laboratory trial—was excluded from review, as there is higher variability in study design among such studies than in clinical case reports.
- **b)** The “patient” from which the bacterium of interest is identified must be human and may be of any age or gender. More than one patient can be included in an emergence event if all patients fell ill and were confirmed to be infected with a resistant bacterium at the same time, for example in the early stages of an outbreak.
- **c)** “Novel” indicates that resistance to a given antimicrobial treatment in the bacterial species in question has not previously been detected or described in the country in question. In this definition, new mechanisms of resistance of a given bacteria to a given antimicrobial combination do not count as novel resistance (e.g. a novel plasmid carrying beta-lactamase in a bacterial species with previously described beta-lactam resistance).
- **d)** “Resistance” is the ability of the bacteria in question to survive standard antibacterial treatment against it. Survival is measured by the bacteria’s ability to continue to cause disease in its host or to spread to others for longer than the standard period following treatment. Standard treatments may be determined by the WHO or by the country in which the resistance event occurred (we deferred to the authors’ selection of standard treatment protocol).
- **e)** An “antimicrobial drug or drug combination” is a drug, drug class, or specific combination of drugs used to treat bacterial infections in humans. We focused exclusively on antibiotic resistance rather than resistance to other antimicrobials due to better data availability and based on the hypothesis that drivers for other antimicrobials might be different than for antibiotics.
- **f)** “Bacteria” is a microorganism that may cause a disease in the domain Bacteria. We did not include cases of resistance in viruses, fungi, or other pathogen types.

For results from the PubMed and Embase search, we programmatically downloaded abstracts and metadata. Article abstracts were manually reviewed by a team of screeners (all authors, see *Author contributions*) to determine whether they likely contained a report of a novel emergence event, according to the above criteria. To ensure uniformity in abstract evaluation, all reviewers received training on the inclusion criteria and were required to achieve 90% agreement with a practice set of 100 previously-screened abstracts. Abstracts were screened separately by two individuals. Reviewers classified articles as “yes”, “maybe”, or “no” for inclusion. If both reviewers classified an article as “yes” or “maybe”, the article was downloaded as full-text for further review and to be coded for the database. In cases when an article was marked as “no” by one of the reviewers, and “yes” by the other reviewer, a third reviewer was assigned to determine if the article should be included. The inter-scorer agreement rate was 82% (i.e., 18% of articles were decided by a third reviewer). 1,583 articles from PubMed and Embase passed review criteria and were downloaded for further review. The R package metagear ^9^ was used to screen articles and manage screening data.

Full texts were downloaded for ProMED-mail search results, as ProMED-mail reports do not have abstracts. The first lines of each report were screened separately by two reviewers. If either reviewer classified a text as “yes” or “maybe”, it was selected for further review and to be coded for the database. 208 ProMED-mail texts passed this screening, contributing to a total of 1,791 total articles from PubMed, Embase, and ProMED-mail selected for review. Further details on the reproducible screening workflow are available in the data repository (see *Data availability*) ^10^.

### Article coding

Articles from PubMed, Embase and ProMED-mail that passed screening were selected for full-text analysis, each by one reviewer, unless quality assurance checks required follow-up review (see *Data cleaning*). Reviewers read full-text articles to determine whether they fully met the case criteria, above, and to extract data by coding the text. Articles were excluded at this stage if it was found that they referred to or were duplicate reports of a previous emergence of the same drug-bacteria resistance within the country, if they reported on a non-bacterial pathogen, or if they did not identify the drug or bacterial species. A total of 294 articles were retained after full-text screening.

Articles were coded for four required fields: study country, drug name, bacteria, and event start date. Drugs were coded to the lowest available taxonomic rank (i.e., specific drug rather than drug class, when available). Similarly, bacteria were coded at the species level when available. Where articles did not include an emergence location or start date, location was inferred from the location of study authors’ institutions (often hospitals), and publication year was used for start date.

In addition to the required fields, articles were coded for the following secondary fields when available: patient attributes (age, gender, country of residence, recent travel locations, symptoms, comorbidities, and outcome), bacterial strains and markers, drug minimum inhibitory concentration (MIC) values, and hospital location (city, state/province). Screeners used MAXQDA ^11^ software for article coding. To our knowledge, open-source equivalents to MAXQDA are not available; however, open-source software such as qcoder ^12^ could be used to replicate MAXQDA if combined with suitable PDF pre-processing steps.

### Data cleaning

We matched free-form values coded in article text to standardized values and ontologies. All locations were matched to Google Places names and geocoded. Country names were maintained according to article reporting and were not standardized to official country recognitions (e.g., United Nations member states). Dates were stored using the international standard ISO 8601. Drug names and bacteria were matched and standardized against the Medical Subject Headings (MeSH) ^13^ and National Center for Biotechnology Information (NCBI) Organismal Classification ^14^ ontologies, respectively, as provided by the Bioportal platform ^15^. We also explored standardizing drug names to the Anatomical Therapeutic Chemical (ATC) ontology, and provide this as an alternative classification ^16^. (We found that ATC classification resulted in similar quantitative results to MeSH-based classification; for example, there are 1,753 events in the ATC-based database versus 1,757 in the MeSH-based database.) Where reported names did not exactly match ontologies or had ambiguous matches, we manually reviewed and corrected names to match the ontologies, in some cases requiring review of the original study to confirm accuracy. Other fields that are not essential to defining emergence events (e.g., patient demographics, comorbidities) were not standardized in the current database release.

Data cleaning and standardization was performed in R version 3.6.1 ^17^, using the tidyverse framework ^18^ for data manipulation. Geocoding used the Google Geocoding API via the ggmap R package ^19^. Dates were standardized using the lubridate R package ^20^.

We implemented quality assurance checks throughout the data processing pipeline. We checked for errors in the MAXQDA article coding by confirming that all values were labeled and that links between values were properly assigned (e.g., links between drug names and MIC values). We checked for any studies missing study location, event start date, drug, or bacteria, and manually revisited these articles to confirm missing fields. We also investigated any study reporting more than one location and/or start date to confirm whether the study described multiple emergence events.

An earlier version of this article can be found on medRxiv (doi: https://doi.org/10.1101/2020.08.13.20165852).

## Results/Discussion

We present a database of 1,757 records of first clinical reports of unique bacterial-drug AMR detections by country, ranging from 1998 to 2017, drawn from 268 peer-reviewed articles and 26 disease surveillance reports (294 total). (While the ProMED-mail search extended to 1994, the first reported event that met our study criteria occurred in 1998.) ProMED-mail reports had similar coverage to the peer-reviewed articles, with events reported in six continents from 1999 through 2016. This database—when used with sufficient consideration of reporting biases (see *Database usage notes*)—can serve as a complement to existing databases that track resistance and spread to support researchers in targeting efforts for surveillance and interventions and analyzing factors that contribute to AMR emergence.

Database materials are available at DOI 10.5281/zenodo.4924992 (see *Data availability*) ^10^. The database can be downloaded as `events-db.csv`. Field names and descriptions from the database are detailed in Table 1. The file `data-processed/articles-db.csv` contains metadata about each article in the database, including citation information and full abstracts. This file can be joined with `events-db.csv` by the `study_id` field.

**Table 1.** Database fields and descriptions.

Field	Description
`study_id`	Unique study identification number that can be joined with `articles_db` for study metadata
`study_country`	Name of country where event occurred. Note that there are some studies that report on events in multiple countries.
`study_iso3c`	Three letter International Organization for Standardization (ISO) code
`study_location`	Full study location (including hospital, city, and state if available)
`study_location_basis`	Spatial basis of study location (e.g., "hospital, city, state_province_district, country")
`residence_location`	Location of patient residence
`travel_location`	Patient travel locations, if any reported. Multiple locations are separated by `;`.
`drug`	Antimicrobial drug, standardized to the Medical Subject Headings (MeSH) ontology ^13^.
`drug_rank`	Taxonomic classification of drug (i.e., drug name or group)
`drug_parent_name`	Name of the taxonomic parent of antimicrobial drug, standardized to the Medical Subject Headings (MeSH) ontology ^13^.
`bacteria`	Name of resistant bacteria, standardized to NCBI Organismal Classification ontology ^14^.
`bacteria_rank`	Taxonomic classification of bacteria name (e.g., “species”, “genus”)
`bacteria_parent_name`	Name of the taxonomic parent of bacteria, standardized to NCBI Organismal Classification ontology ^14^.
`bacteria_parent_rank`	Taxonomic classification of bacteria parent name (e.g., “species”, “genus”)
`start_date`	Date that patient was presented to hospital in format of *yyyy-mm-dd*
`start_date_rank`	Specificity of the start date (i.e., year, month, day)
`end_date`	Date that patient was released from hospital or died in format of *yyyy-mm-dd*
`data_source`	Whether data source is ‘peer-reviewed study’ or ‘promed-mail report’

In addition to the repository, we present intermediate data used in the process of database development in our GitHub repository { https://github.com/ecohealthalliance/amr-db }. All abstracts and ProMED-mail reports that were screened are in the `screening` directory. Raw exports from coded full-text articles are in .xlsx form in `data-raw/coded-segments`. (The coded full text articles themselves are not available in the data repository.) A pre-filtered, pre-transformed database that contains all fields (primary and secondary) and events (including some non-emergent events) is in `data-processed/segments.csv`. An alternative version of the database with drug names standardized to the ATC ontology is available in the `alt-db-atc/` directory, and a one-to-one comparison of MeSH and ATC drug names from the database is available in `figures-and-tables/mesh-atc-comparison.csv`. Further details on directory structure and usage are provided in the data repository documentation.

**Figure 2.**
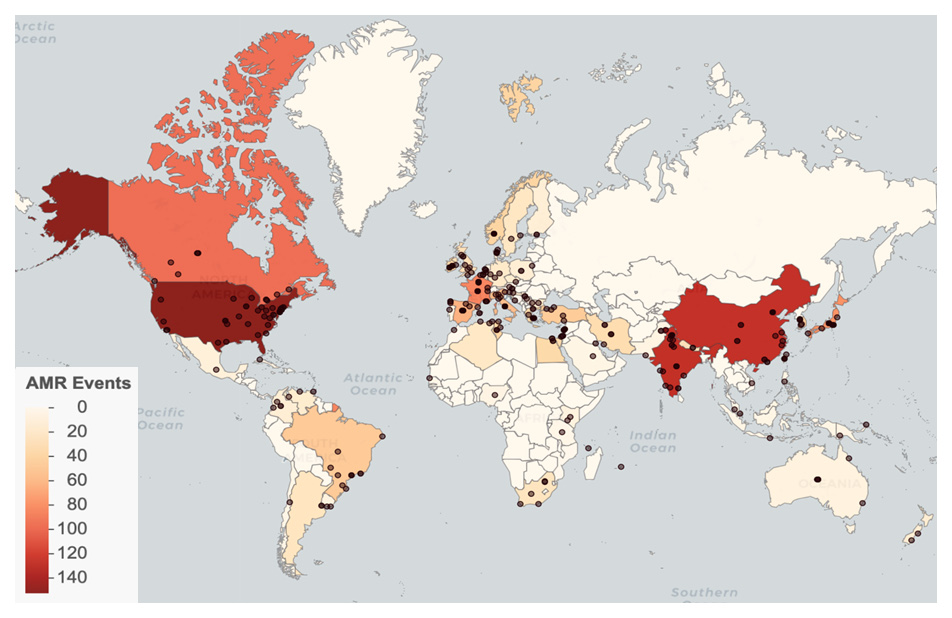
Global distribution of antimicrobial resistance (AMR) emergence events. Points represent locations. Countries are shaded by event count.

**Figure 3.**
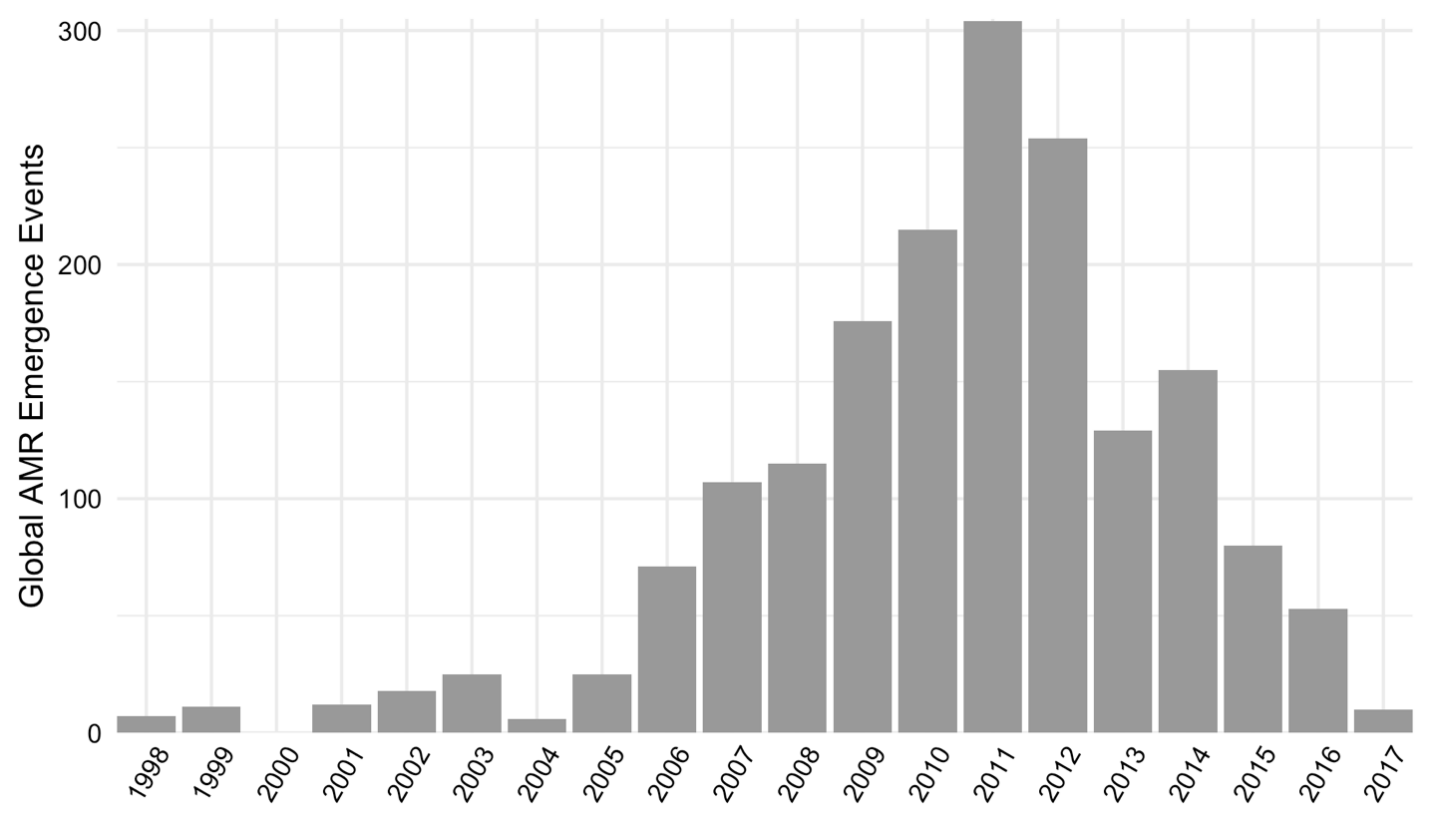
Global number of antimicrobial resistance (AMR) emergence events in the database disaggregated by year.

We identified AMR emergence events in 66 countries, with the most reported events in the United States (152), China (128), and India (127). ( Figure 2). Events were reported from 1998 through 2017, with the greatest number of reported events occurring in 2011 ( Figure 3). See *Database usage notes* for discussion on the effects of reporting bias on the spatial and temporal coverage of the database.

**Figure 4.**
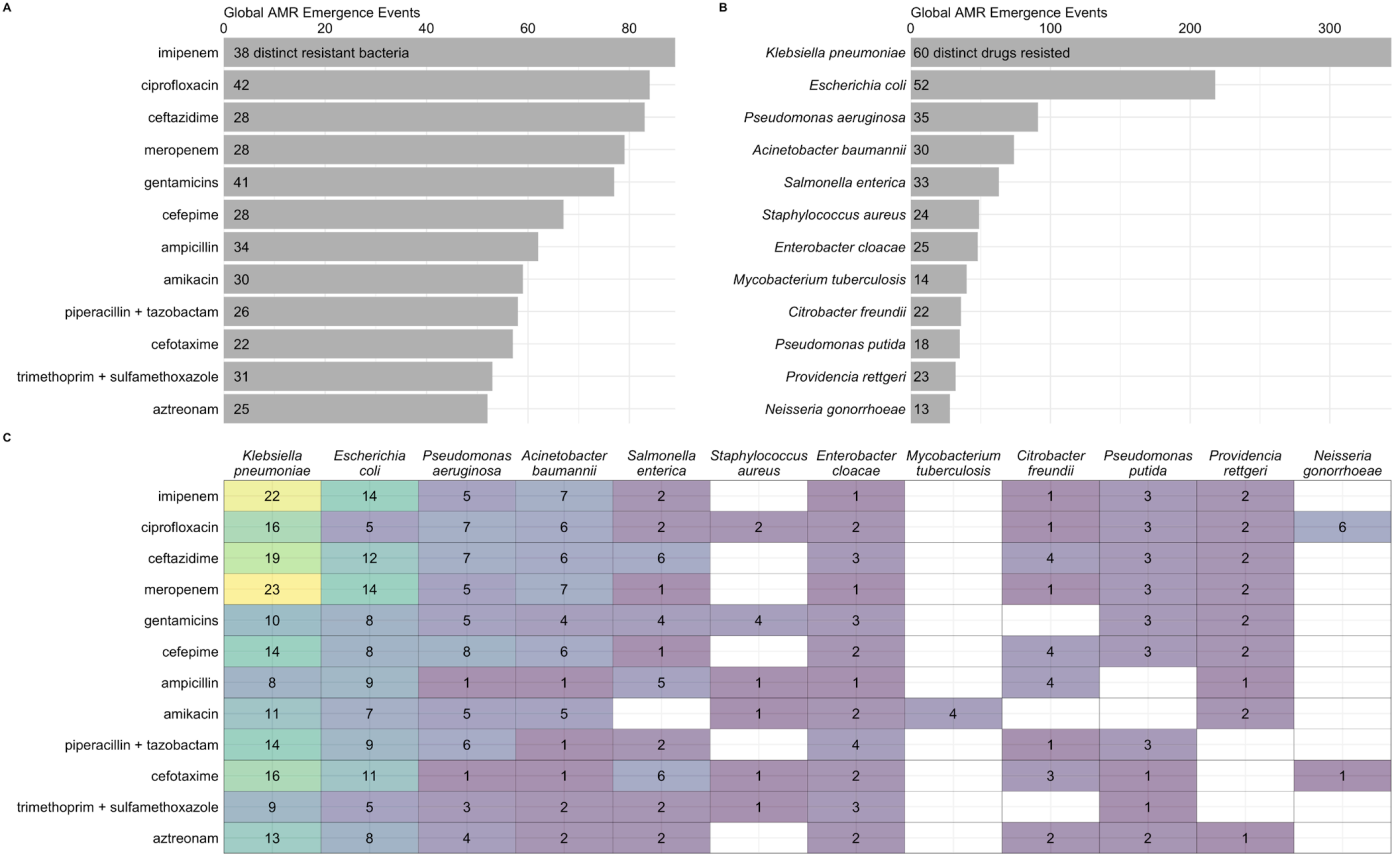
Global antimicrobial resistance (AMR) emergence events for top 12 drugs ( **a**) and bacteria ( **b**), and for their combinations ( **c**). Counts within bars represent distinct number of resistant bacteria ( **a**) and drugs ( **b**).

We found that *Klebsiella pneumoniae* and *Escherichia coli* were the most common bacteria in emergence events ( Figure 4), supporting results from other databases that have found high rates of AMR prevalence for these species ^3, 8^. Of concern, our database indicates that both bacteria species had the greatest number of reports of novel resistance to imipenem and meropenem ( Figure 4), which are carbapenem antibiotics that are considered critical for treating infections acquired in health care settings ^3, 21, 22^. Also concerning were 23 cases of emergent resistance to the antibiotic colistin in 16 countries and 14 distinct bacteria, including *K. pneumoniae* and *E. coli.* Colistin is critically important for treating infections when no other options are available ^22, 23^.

Further analysis is needed to understand the underlying causes of AMR emergence, including the relative contributions of human and livestock antibiotic consumption. In addition to antibiotic consumption, risk factors for emergence may include human travel and migration; country population, GDP, and spending on healthcare; and production of antibiotics. Identifying risk factors for AMR emergence will support improved AMR surveillance and interventions and will align with a “One Health” approach of linking drivers from the human, animal, and environmental sectors ^24^.

Information in this repository can be used to inform international initiatives aimed to reduce and prevent the emergence of AMR. For example, the WHO Global Action Plan on AMR articulates the need for country capacity to detect emergence of resistance ^1^. International organizations are developing tracking systems that seek to enhance surveillance and reporting at the global level (e.g., the Global Antimicrobial Resistance and Use Surveillance System [ GLASS]). Long-term, the AMR digital detection interface launched in 2020 through the Program for Monitoring Emerging Diseases (ProMED) may collate future emergence events and can consider antimicrobial use and other relevant metadata examined in this study to support trend analysis ^25^. Finally, improved attention to AMR emergence is in line with the prevention-oriented focus of the Action Package on AMR under the Global Health Security Agenda, a partnership of 70+ countries working to strengthen capacities to make the world safe against infectious disease threats.

## Database usage notes

The database is developed using only English-language scientific literature and medical reporting events. It therefore represents events reported in this limited subset rather than truly representative clinical cases and strongly reflects reporting effort and practices. It is likely that AMR emergence events are systematically missing from the database for countries that are not English speaking and/or have less health care monitoring and reporting capacity, including those with lower GDP. Therefore, it is imperative that any analysis of the data account for the effects of reporting bias ^26^. In addition, future efforts to characterize AMR emergence may consider other sources of data including gray literature and government reports. These sources of data may be particularly useful for informing fine-scale data needs for country decision making.

Our review did not include any quality assessments of the published scientific literature and disease surveillance reports. Data sourced from the peer-reviewed literature has gone through a process of validation and quality checked by the journal editors and reviewers. ProMED-mail reports, which are generated in real-time, are screened by regional moderators to ensure data validity, but no peer-review or editorial process is involved. This is a potential source of uncertainty in our database, and is indicated in the column ‘data_source`.

Temporal patterns of AMR emergence events in the database reflect potentially incomplete coverage prior to 2006, as only ProMED-mail (not PubMed or Embase) was searched for this period. Further, the decrease in emergence events following the peak in 2011 likely reflects, in part, lags between event occurrence and reporting. Continued updating of the database is needed to determine long-term temporal trends.

There are several sources of ambiguity in the dataset due to imprecise reporting in literature ( Table 2). While most studies reported the exact city or hospital of the emergence event (n = 200), others reported only state/province (n = 21) or study country (n = 73). Differences in geographic scales would need to be considered if using this dataset for spatial analysis. In addition, there are inconsistencies in studies’ reporting of drug and bacteria names. While most studies reported specific drug names (n = 83), others reported broad classes of drugs (n = 32). Study authors that reported classes of drugs may have administered multiple drugs to the patient(s), but because the specific drugs were not reported, we were only able to count the drug as part of a single event. In addition, some drugs may be been administered as part of a mixture whereas other drugs were administered independently of each other (e.g., following failure of previous drug to treat infection). Due to ambiguity in reporting, we were not able to parse out mixtures versus independent drugs, and therefore treated each drug reported as independent. Bacteria were primarily reported to the species level (n = 106), but some were reported only to the genus or family level (n = 5). In these cases, it is possible that the species was novel or not identifiable. Many studies that reported bacterial species also reported strains, and it is possible that there are differences in resistance by strain. However, due to inconsistent reporting, we have not standardized reported strains.

**Table 2.** Classification specificity of four primary database fields.

	Classification	Count
**Drug**	drug name	83
	drug group	32
	drug group/name combo	3
**Bacteria**	species	106
	genus	4
	family	1
**Location**	hospital	123
	city	77
	state/province/district	21
	country	73
**Start Date**	day	47
	month	121
	year	134

This database presents first AMR emergence events aggregated at the country level. Emergence events may be due to either local mutation events or imported resistance from other geographies. Future analyses examining the underlying determinants of first emergence at a country level should consider import as a possible mechanism. For example, some studies reported locations of residence and recent travel of patients, and where available we have included this information, which may support such analysis. Analyses only examining first global emergence events should remove records of all but the earliest dates of each unique bacteria-drug combination.

## Data availability

Zenodo: A global repository of novel antimicrobial emergence events. http://doi.org/10.5281/zenodo.4924992 ^10^.

This project contains the following underlying data:

• `events-db.csv` (AMR emergence events database)
• `data-processed/articles-db.csv` (Metadata about each article in the database, including citation information and full abstracts. This file can be joined with `events-db.csv` by the `study_id` field.)
• `screening/` directory (All abstracts and ProMED-mail reports that were screened for potential inclusion in the database)
• `data-raw/coded-segments` (raw exports from coded full-text articles)
• `data-processed/segments.csv` (A pre-filtered, pre-transformed database that contains all fields ad events)

License: MIT

## Code availability

Source code available from: https://github.com/ecohealthalliance/amr-db

Archived source code at time of publication: http://doi.org/10.5281/zenodo.4924992 ^10^

License: MIT
