# Supplementary material for: A global repository of novel antimicrobial emergence events
Source: F1000Res. 2021 Jun 21;9:1320. Originally published 2020 Nov 12. [Version 2] doi: 10.12688/f1000research.26870.2 (PMC8596184; doi:10.12688/f1000research.26870.2)
Supplement: Peer review history

# Peer review: Reviewer response for version 2

**DOI:** 10.5256/f1000research.57747.r99191

**Published:** 2021-11-16

**Authors:** Jorge Pinto Ferreira

**Affiliations:** 1 Antimicrobial Resistance and Veterinary Products Department, World Organisation for Animal Health, Paris, France

1. The title needs to reflect that the paper only focus on humans.
2. "Novel" in this case only refers to "novel in the country" - this is a major characteristic, that needs to be emphasized, if possible, even in the title.
3. In the introduction, the reference to IACG does not make sense any more, as the IACG is no longer existent. It lead to a couple of new structures, including, for example, the Global Leaders Group, and these should be mentioned in the paper.
4. Fig. 3: even if speculative, the peaks between 2010-2012 should be explained.

Are sufficient details of methods and materials provided to allow replication by others?

Yes

Is the rationale for creating the dataset(s) clearly described?

Yes

Are the datasets clearly presented in a useable and accessible format?

Yes

Are the protocols appropriate and is the work technically sound?

Yes

Reviewer Expertise:

AMR, AMU, policy

I confirm that I have read this submission and believe that I have an appropriate level of expertise to confirm that it is of an acceptable scientific standard, however I have significant reservations, as outlined above.

# Peer review: Reviewer response for version 2

**DOI:** 10.5256/f1000research.57747.r87972

**Published:** 2021-08-09

**Authors:** Sergey Eremin

**Affiliations:** 1 World Health Organization, Geneva, Switzerland

We have no further comments to make.

Are sufficient details of methods and materials provided to allow replication by others?

Partly

Is the rationale for creating the dataset(s) clearly described?

Partly

Are the datasets clearly presented in a useable and accessible format?

Partly

Are the protocols appropriate and is the work technically sound?

Partly

Reviewer Expertise:

Surveillance of antimicrobial resistance.

I confirm that I have read this submission and believe that I have an appropriate level of expertise to confirm that it is of an acceptable scientific standard.

# Peer review: Reviewer response for version 1

**DOI:** 10.5256/f1000research.29674.r78874

**Published:** 2021-02-15

**Authors:** Ellen Stobberingh

**Affiliations:** 1 Care And Public Health Research Institute (CAPHRI), Maastricht University Medical Centre (MUMC), Maastricht, The Netherlands

The authors analysed the prevalence of novel AMR in humans in the country. Several questions need to be clarified:

 - Inclusion criteria: 

- AMR in asymptomatic commensals were not included as these do not meet the inclusion criteria of clinical cases. However, resistance in commensals is frequently the precursor of resistance in clincal isolates. Excluding the commensals might contribute to lower prevalence and later report of resistance.
- Focus on humans only did not address the One health approach which is especially relevant in AMR because of the spread from animals to humans. This should be added in the discussion.
- Novel: was defined as novel for that country. But the resistance might be described already in other countries. Transfer from these countries for instance via travel might contribute to spread of these AMR to other countries and is not a novel resistance. Pick up of AMR resistance during travel starts mostly as a commensal and later on possible as infection. Please comment.
- Resistance: defined as to survive AMR treatment, i.e. the bacteria *is able to continue to cause disease in the host or to spread to others for a longer than the standard period as determined by the WHO or the national guidelines.* What to do in case of discrepancy between these two?
- Interscore 82% in case of discrepancy the decision of a third reviewer was decisive?
- What is the possible explanation for the highest prevalence in 2011?
- Resistance to carbapenems as last resort, colistin when no other options are available. This suggests a discrepancy, please rephrase.

Are sufficient details of methods and materials provided to allow replication by others?

Yes

Is the rationale for creating the dataset(s) clearly described?

Partly

Are the datasets clearly presented in a useable and accessible format?

Yes

Are the protocols appropriate and is the work technically sound?

Partly

Reviewer Expertise:

Antimicrobial resistance prevalence and spread, Bacteriology.

I confirm that I have read this submission and believe that I have an appropriate level of expertise to confirm that it is of an acceptable scientific standard, however I have significant reservations, as outlined above.

# Response

**Published:** 2021-06-10

**Authors:** Emma Mendelsohn

**Affiliations:** EcoHealth Alliance, USA

Thank you for your thorough review and comments (especially appreciate your thoughts on the One Health context and potential spread mechanisms). We address your comments below. 

****

**AMR in asymptomatic commensals were not included as these do not meet the inclusion criteria of clinical cases. However, resistance in commensals is frequently the precursor of resistance in clincal isolates. Excluding the commensals might contribute to lower prevalence and later report of resistance.**

****

 We agree that asymptomatic commensals are important to identifying resistance before clinical emergence. However, we did not include commensals in the database as we found there is high variability in study design among these studies (e.g., drugs tested). Clinical reports, while still subject to variability and bias, tend to be more consistent in design (reporting only on resistances observed with illness). We address this concern in the *Abstract and report screening* section of the manuscript.

****

**Focus on humans only did not address the One health approach which is especially relevant in AMR because of the spread from animals to humans. This should be added in the discussion.**

****

 This is a good point and we have added text about potential risk factors (including livestock) for emergence and the relevance to One Health in the discussion section.

****

**Novel: was defined as novel for that country. But the resistance might be described already in other countries. Transfer from these countries for instance via travel might contribute to spread of these AMR to other countries and is not a novel resistance. Pick up of AMR resistance during travel starts mostly as a commensal and later on possible as infection. Please comment.**

****

 We agree and note that emergence events within a country may be due to mutation or transport from other geographies. For first global emergence events, users of the database should filter to the first occurrence of each bacteria-drug combination. We have added discussion about travel and migration being potential risk factors for emergence in countries.

****

**Resistance: defined as to survive AMR treatment, i.e. the bacteria is able to continue to cause disease in the host or to spread to others for a longer than the standard period as determined by the WHO or the national guidelines. What to do in case of discrepancy between these two?**

****

 As most authors did not report which guidelines they used, we deferred to the authors selection of protocols and did not evaluate any potential discrepancies. We have added text to clarify this.

****

**Interscore 82% in case of discrepancy the decision of a third reviewer was decisive?**

****

 Yes; we have rephrased to clarify this.

****

**What is the possible explanation for the highest prevalence in 2011?**

****

 We have rephrased the text in *Data usage notes* to clarify that the peak in 2011 is likely an effect of reporting lag for more recent events.

****

**Resistance to carbapenems as last resort, colistin when no other options are available. This suggests a discrepancy, please rephrase.**

****

 Good catch. We have rephrased.

# Peer review: Reviewer response for version 1

**DOI:** 10.5256/f1000research.29674.r75116

**Published:** 2020-12-07

**Authors:** Sergey Eremin, Barbara Tornimbene

**Affiliations:** 1 World Health Organization, Geneva, Switzerland

The paper represents an impressive effort to fill in the gaps in our understanding of emergence and spread of antimicrobial resistance in bacterial pathogens and provides results of a systematic review of English-language scientific literature and surveillance reports to identify the spatial and temporal patterns of the emerging AMR.

**Is the rationale for creating the dataset(s) clearly described?**

 Although the author rationale for generating a publicly available source of temporal and spatial data for emerging resistance is clear, it could be better explained in the title and the introduction that the creating of a database is the objective of the study. The authors could also go into more detail on how the database will support on-going agreements and programs, particularly by defining on-going agreements. It would be also important to clarify what will be the role of the government of countries that appear in the dataset, in terms of acknowledging and validating published data.

**Are the protocols appropriate and is the work technically sound?**

 Merging of results originating from peer-reviewed literature, which undergoes a strict scientific scrutiny, and data generated by ProMED-mail, which does not follow the same rigour, can be exposed, particularly when reporting novel events, to high level of inconsistency. No distinction between study designs screened in the literature was applied and results adjusted accordingly. The exclusion criteria could be expanded. For example, no assessment of the quality and reliability of these studies was done before including them in the final pool of articles.

 The search strategy may benefit from inclusion of terms related to “drug resistance”, the term used in quite a number of reports instead of “antibiotic resistance” or “antimicrobial resistance”, especially when reporting infections caused by MDR or XDR or PDR pathogens.

 Finally, the authors mentioned in the “Database usage notes” section that the reported events represent a limited subset of information and strongly reflect reporting effort and practices. Other limitations are also listed in this section. This should probably be part of the discussion and better reflected on the abstract when listing the most common bacteria demonstrating new resistance. Moreover, an in-depth discussion on the impact of these bias should be added, particularly the effect that variability in reporting effort and practices, and quality and reliability of the studies, have on obtained results.

**Are sufficient details of methods and materials provided to allow replication by others?**

 Some definitions in the inclusion criteria are not sufficiently clear.

 A clinical case is defined as “an individual who presents with symptoms to a medical professional and is determined by a medical professional to have been infected with the bacterium in question”. This sounds rather vague and we believe that the authors could be more consistent when defining “bacterium in question” or “bacterium of interest”. This is important to understand how cases were included.

 The definition of novel resistance seems to be uncertain. It has to be clearly explained in the paper why only phenotypic test results were chosen and why only specific drug-bug combinations are considered and not emergence of certain types of multidrug resistance. The authors might also want to clarify why detection of a novel gene responsible, using example given in the paper, for production of a new type of beta-lactamase or, specifically, carbapenemase, is not considered novel and should be ignored. Such an event has to be detected and followed as it may have a significant impact on treatment options and other public health implications. Again, more details should be given on how the “novelty” was identified when reviewing the papers. For example, the paper coded 15378 in the database describes both phenotypic and molecular resistance testing results obtained from 12 clinical isolates from 12 patients with infections caused by *Enterococcus faecium* and 11 supposedly novel resistance events were included in the database. But the only event that was defined by the authors of the report as novel was the first detection in the country of the clonal complex 17 VREF. Resistance to e.g. clindamycin, gentamicin, and several other antibiotics in all reported isolates could hardly be considered novel or emerging. 

 The authors should better explain the added value of including the “drug_parent_name” variable with the names of the taxonomic parent of antimicrobial drug, standardized to the MeSH ontology. While it may be reflecting the methodology of the database creation, using a classification more suited to the field of study, such as e.g. the Anatomical Therapeutic Chemical (ATC) classification system may be considered.

 Finally, as the main goal of the database is to present temporal and spatial data, a better definition of temporal data should be given. In the paper the authors mention “event date”, “start date”, “study date” and “end date”. Aside the lack of consistency it should be clearer if the authors are considering the date the patients started experiencing symptoms, the date the patients was diagnosed, the date the diagnosis results were obtained, or the date the study started. This is key to allow replication by others.

**Are the datasets clearly presented in a useable and accessible format?**

 The detailed dataset is easily accessible and could be downloaded but is not sufficiently user-friendly. Using it would require certain data management skills from the end-users and therefore might limit their number.

Are sufficient details of methods and materials provided to allow replication by others?

Partly

Is the rationale for creating the dataset(s) clearly described?

Partly

Are the datasets clearly presented in a useable and accessible format?

Partly

Are the protocols appropriate and is the work technically sound?

Partly

Reviewer Expertise:

Surveillance of antimicrobial resistance.

We confirm that we have read this submission and believe that we have an appropriate level of expertise to confirm that it is of an acceptable scientific standard, however we have significant reservations, as outlined above.

# Response

**Published:** 2021-06-10

**Authors:** Emma Mendelsohn

**Affiliations:** EcoHealth Alliance, USA

Thank you for your thorough review. We appreciate your attention to detail and thoughtful comments, which we address below. 

**Is the rationale for creating the dataset(s) clearly described?**

**Although the author rationale for generating a publicly available source of temporal and spatial data for emerging resistance is clear, it could be better explained in the title and the introduction that the creating of a database is the objective of the study. The authors could also go into more detail on how the database will support on-going agreements and programs, particularly by defining on-going agreements. It would be also important to clarify what will be the role of the government of countries that appear in the dataset, in terms of acknowledging and validating published data.**

****

 We agree and have emphasized the database as a study objective by changing the title to “A global repository of novel antimicrobial emergence events”. We also added details on the rationale and main goals of the paper in the introduction, highlighting the relevance of this dataset in supporting on-going international agreements and programs.

**Are the protocols appropriate and is the work technically sound?**

**Merging of results originating from peer-reviewed literature, which undergoes a strict scientific scrutiny, and data generated by ProMED-mail, which does not follow the same rigour, can be exposed, particularly when reporting novel events, to high level of inconsistency. No distinction between study designs screened in the literature was applied and results adjusted accordingly. The exclusion criteria could be expanded. For example, no assessment of the quality and reliability of these studies was done before including them in the final pool of articles.**

****

 Thank you for the relevant comment. This is an important point to be considered by the end-users. We have clarified in the manuscript that we did not conduct an assessment of study/report quality and have described this as a source of uncertainty. In addition, we have added a field to the database indicating whether an event is from a published study or from ProMED-mail, and we include in the text summarized counts of events by source type.

****

**The search strategy may benefit from inclusion of terms related to “drug resistance”, the term used in quite a number of reports instead of “antibiotic resistance” or “antimicrobial resistance”, especially when reporting infections caused by MDR or XDR or PDR pathogens.**

****

 We agree that “drug resistance” may yield relevant results. Unfortunately we are unable to redo the existing literature search at this time due to the high level of effort required and additional human resources not available at this time. We will include this term in future iterations of the project.

****

**Finally, the authors mentioned in the “Database usage notes” section that the reported events represent a limited subset of information and strongly reflect reporting effort and practices. Other limitations are also listed in this section. This should probably be part of the discussion and better reflected on the abstract when listing the most common bacteria demonstrating new resistance. Moreover, an in-depth discussion on the impact of these bias should be added, particularly the effect that variability in reporting effort and practices, and quality and reliability of the studies, have on obtained results.**

****

 We thank the reviewer for the suggestion. Bias in reporting effort is indeed a recurrent problem in surveillance. We have added mention of reporting biases to the abstract and discussion section (referring readers to Database usage notes for further detail). We also now address quality of studies as a source of uncertainty in the database.

****

**Are sufficient details of methods and materials provided to allow replication by others?**

**Some definitions in the inclusion criteria are not sufficiently clear.**

****

**A clinical case is defined as “an individual who presents with symptoms to a medical professional and is determined by a medical professional to have been infected with the bacterium in question”. This sounds rather vague and we believe that the authors could be more consistent when defining “bacterium in question” or “bacterium of interest”. This is important to understand how cases were included.**

****

 We adjusted the wording to clarify that bacteria is any species of pathogen in the domain Bacteria, and have replaced the term “bacterium in question” with “bacterium species reported”.

****

 Due to inconsistencies in reporting, we were unable to consistently identify pathogens by strain. However, this data is available as an unstandardized field in the database and we added .

****

**The definition of novel resistance seems to be uncertain. It has to be clearly explained in the paper why only phenotypic test results were chosen and why only specific drug-bug combinations are considered and not emergence of certain types of multidrug resistance. The authors might also want to clarify why detection of a novel gene responsible, using example given in the paper, for production of a new type of beta-lactamase or, specifically, carbapenemase, is not considered novel and should be ignored. Such an event has to be detected and followed as it may have a significant impact on treatment options and other public health implications. Again, more details should be given on how the “novelty” was identified when reviewing the papers. For example, the paper coded 15378 in the database describes both phenotypic and molecular resistance testing results obtained from 12 clinical isolates from 12 patients with infections caused by Enterococcus faecium and 11 supposedly novel resistance events were included in the database. But the only event that was defined by the authors of the report as novel was the first detection in the country of the clonal complex 17 VREF. Resistance to e.g. clindamycin, gentamicin, and several other antibiotics in all reported isolates could hardly be considered novel or emerging.**

****

 We agree this is a source of uncertainty in the database. In response to your comment, we have revisited the drug name standardization and disaggregated all drug combinations, as we had not been consistent in identifying combinations. We are now treating all drugs as being separately administered. Unfortunately, due to inconsistencies in reporting, we are not able to effectively characterize which drugs were administered independently and which were administered as part of a complex. We address this uncertainty in the Database usage notes.

****

**The authors should better explain the added value of including the “drug_parent_name” variable with the names of the taxonomic parent of antimicrobial drug, standardized to the MeSH ontology. While it may be reflecting the methodology of the database creation, using a classification more suited to the field of study, such as e.g. the Anatomical Therapeutic Chemical (ATC) classification system may be considered.**

****

 Based on your comments we explored standardizing drug names to ATC. We found that results were very similar to MeSH (i.e., counts changed only slightly) . We discuss this in the manuscript and have added an alternate version of the database with ATC standardization to the project repository.

****

**Finally, as the main goal of the database is to present temporal and spatial data, a better definition of temporal data should be given. In the paper the authors mention “event date”, “start date”, “study date” and “end date”. Aside the lack of consistency it should be clearer if the authors are considering the date the patients started experiencing symptoms, the date the patients was diagnosed, the date the diagnosis results were obtained, or the date the study started. This is key to allow replication by others.**

****

 We switched to consistent usage of “start date” and clarified that it refers to the date the patient presented to the hospital or clinic.

****

**Are the datasets clearly presented in a useable and accessible format?**

**The detailed dataset is easily accessible and could be downloaded but is not sufficiently user-friendly. Using it would require certain data management skills from the end-users and therefore might limit their number.**

****

 We added a sentence to clarify that the database is available for download as a single csv file (`events-db.csv`)
